# Supplementary material for: Krill and salp faecal pellets contribute equally to the carbon flux at the Antarctic Peninsula
Source: Nat Commun. 2021 Dec 9;12:7168. doi: 10.1038/s41467-021-27436-9 (PMC8660819; doi:10.1038/s41467-021-27436-9)
Supplement: Supplementary file 1 — Supplementary Information [file 41467_2021_27436_MOESM1_ESM.pdf]

# Supplementary Information for Krill and salp faecal pellets contribute equally to the carbon flux at the Antarctic Peninsula

Pauli et al.

Supplementary Discussion

The POC to volume ratio of salp faecal pellets determined in this study matches previous measurements[1], while the ratio for krill faecal pellets (0.08 mg C mm^{-3}) was slightly higher than that reported in previous studies (0.02–0.06 mg C mm^{-3})[2,3]. The POC to volume ratio of krill pellet was determined based on six replicate filters with about ten pellets on each filter. The carbon content of faecal pellets varies with season, food quality and quantity, as well as with the digestion and assimilation efficiency of the grazing zooplankton organism. In another study, the composition of krill faecal pellets from the same cruise was analysed using 18S metabarcoding, and showed a high content of silicified flagellates (*Ebria* sp.) and diatoms[4]. Both, diatoms and smaller phytoplankton, such as ebridian flagellates, can account for a large proportion of the net primary production, thus channelling high amounts of carbon through the food web[5].

Supplementary Figures and Tables

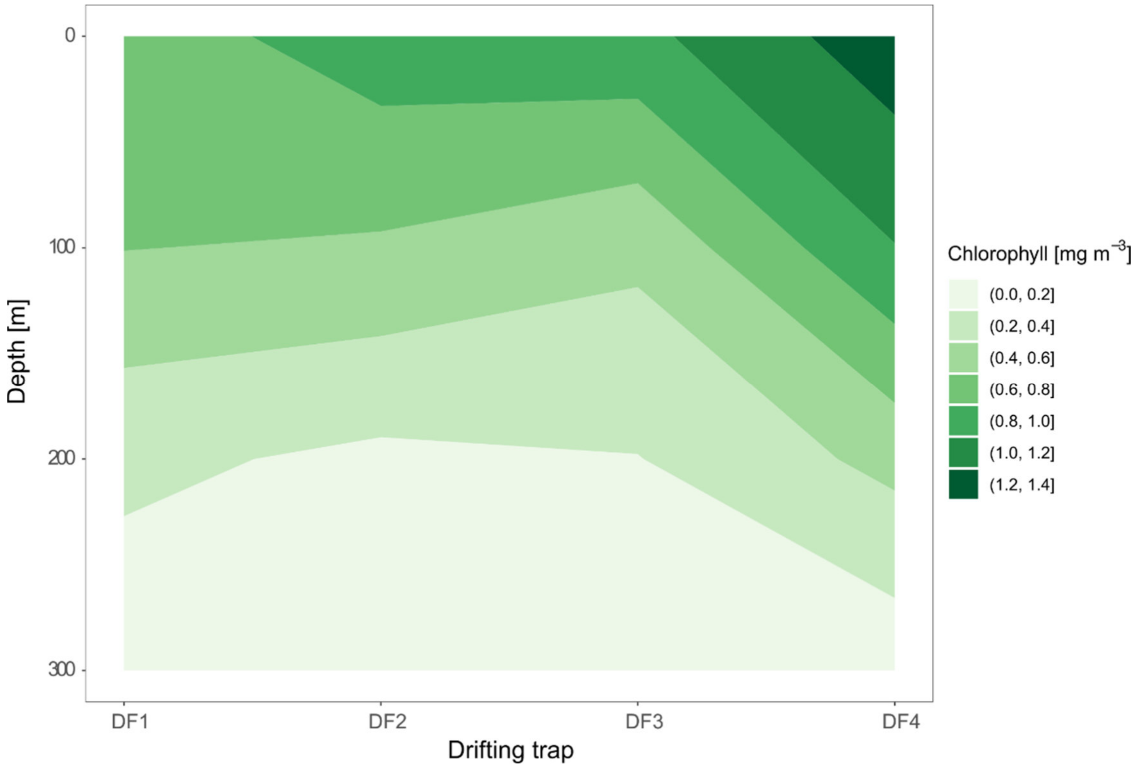

Supplementary Figure 1. Vertical distribution of chlorophyll *a* in mg m^{-3} over the top 300 m of the water column for the drifting trap deployments one to four (DF1 – 4). The different shades of green depict the chlorophyll concentration in steps of 0.2 mg m^{-3}. There are no data available for the period of drifting trap DF 5.

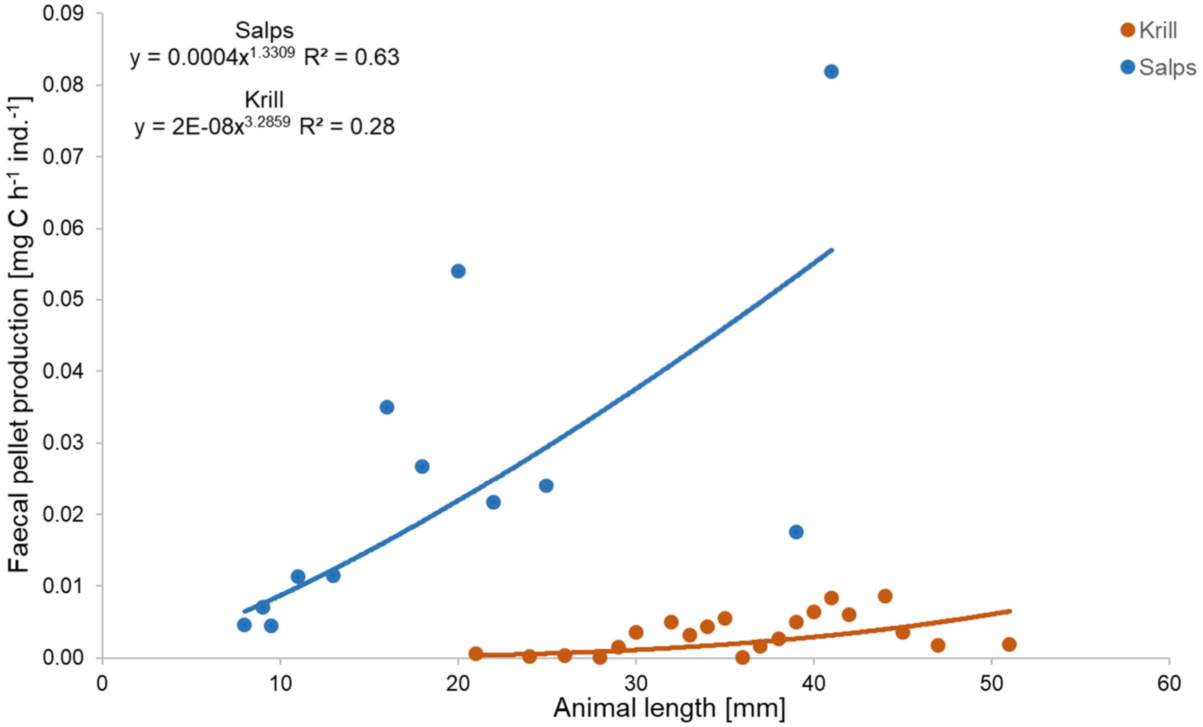

Supplementary Figure 2. Faecal pellet carbon production in mg C h^{-1} $ind.^{-1}$ of krill in red, and salps in blue, as a function animal length in mm. For salps, the oral-atrial length (OAL) was used. Krill length was measured as total length following the AT method by Mauchline (1980). Both regressions are power functions and the respective equations are shown.

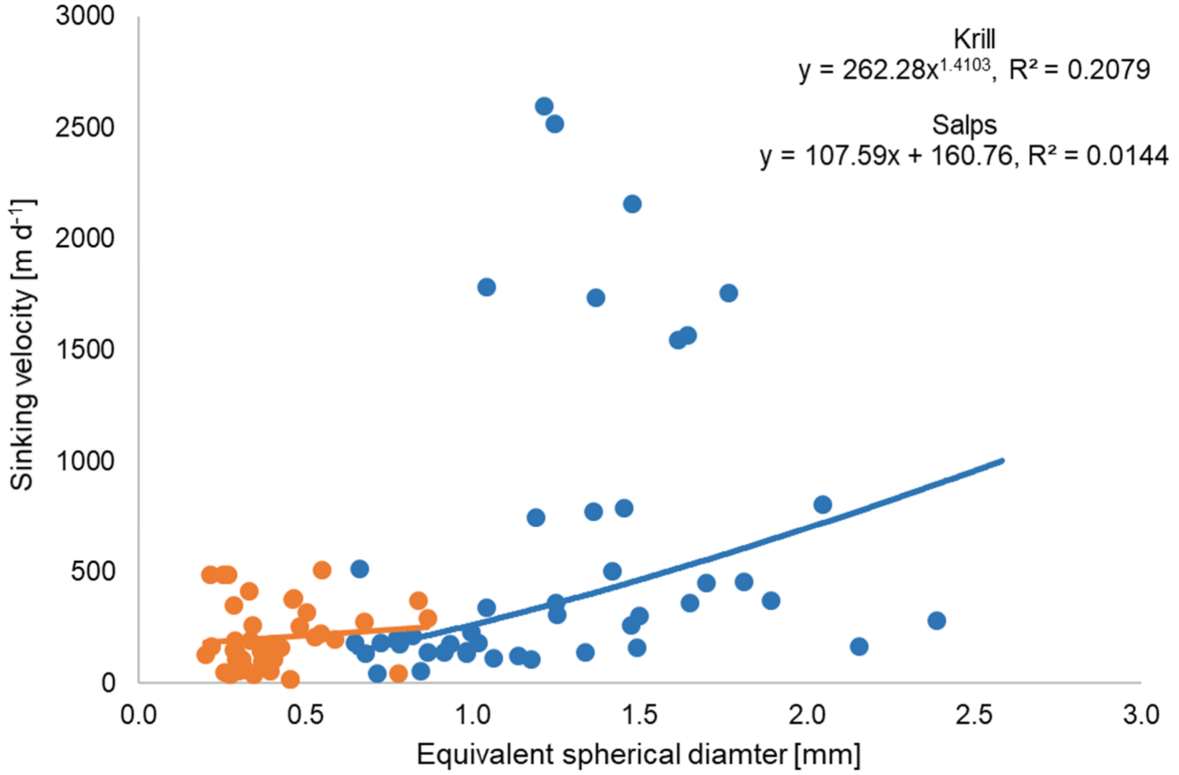

Supplementary Figure 3. Sinking velocity in m d^{-1} for krill (orange) and salp (blue) faecal pellets as a function of pellet size (Equivalent spherical diameter, mm). For krill pellets a power function was plotted, for salp pellets a linear regression is shown.

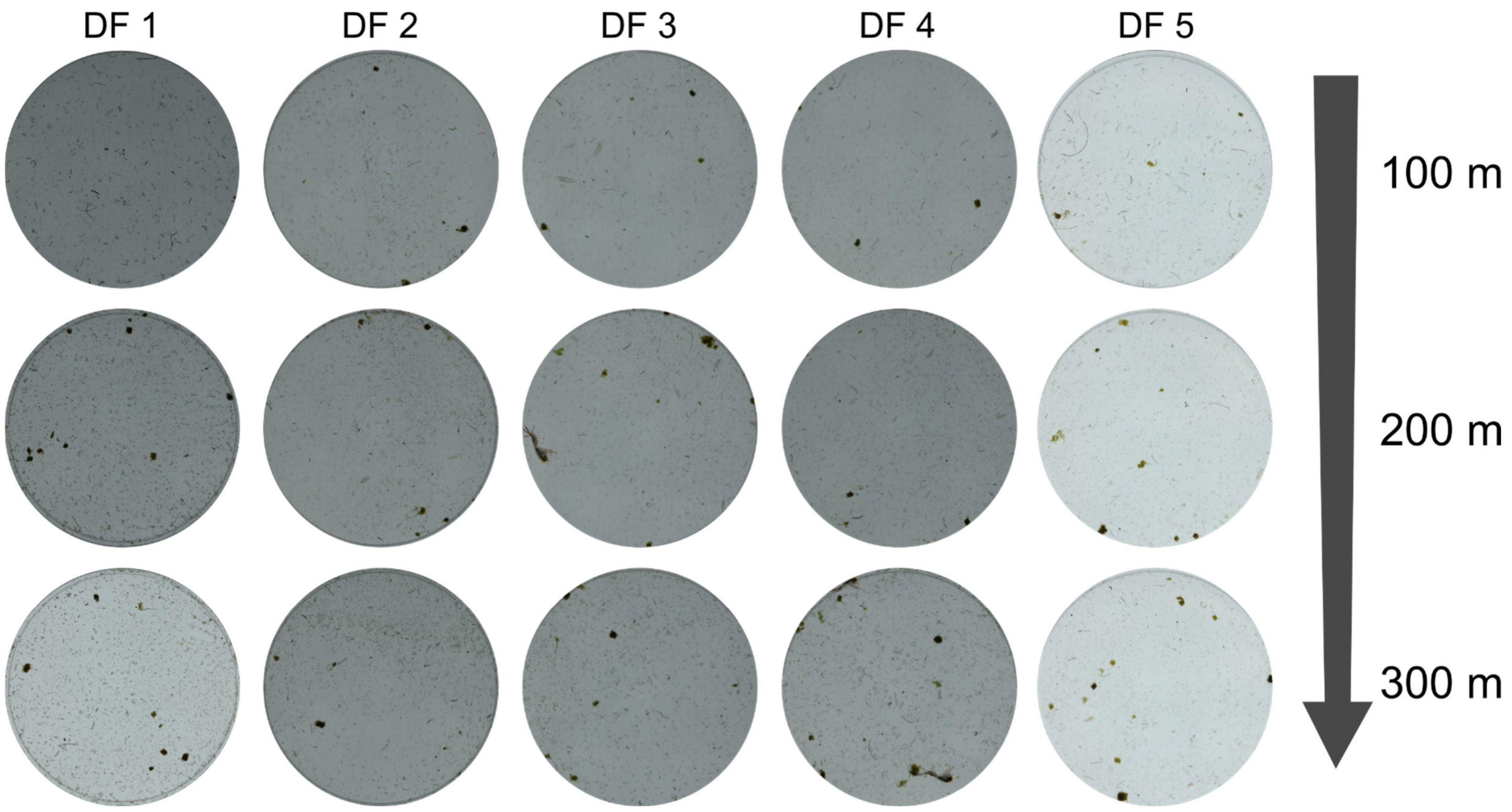

Supplementary Figure 4. Pictures of the gel traps for each deployment depth for each of the five drifting traps (DF 1 – 5). Salp and krill faecal pellets were found in all gel traps. Gels were deployed in one of four collection cylinders per depth and drifting traps to preserve the sinking particles in shape and size. Pictures by M. Iversen, C. Flintrop and N.-C. Pauli.

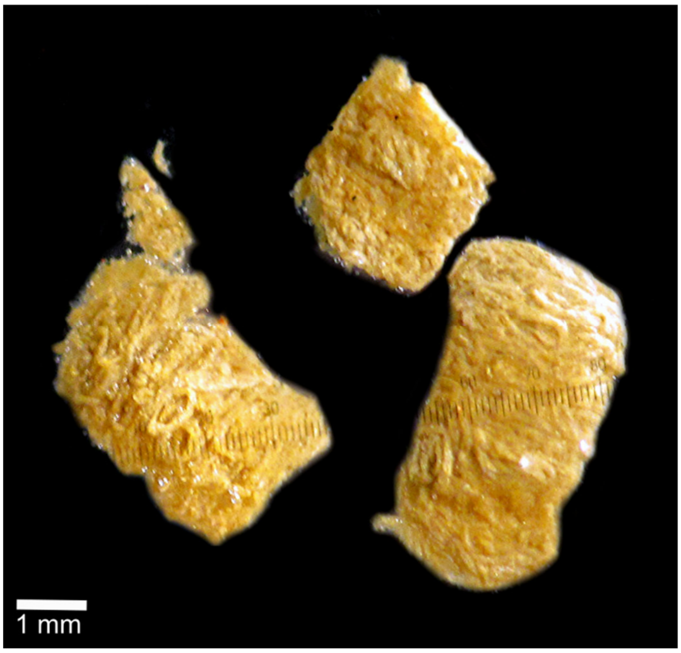

Supplementary Figure 5. Salp faecal pellet containing ingested krill faecal pellets (type 2). Faecal pellets were produced by an aggregate salp with a size of 27 mm (oral-atrial length). Picture by E.A. Pakhomov.

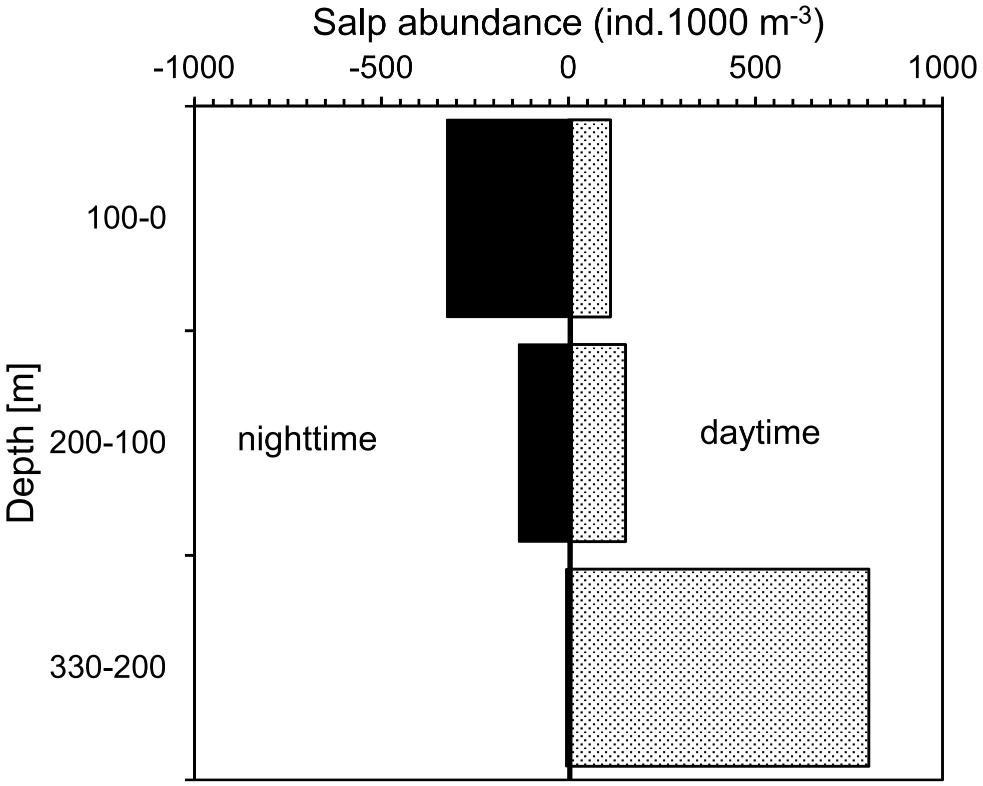

Supplementary Figure 6. Vertical distribution of *Salpa thompsoni* during day (grey) and night (black) from the surface to a depth of 330 m. Vertical distribution was determined from Multi RMT hauls (Rectangular Midwater Trawls) at two stations at Elephant Island in the same time period the five drifting traps were deployed.

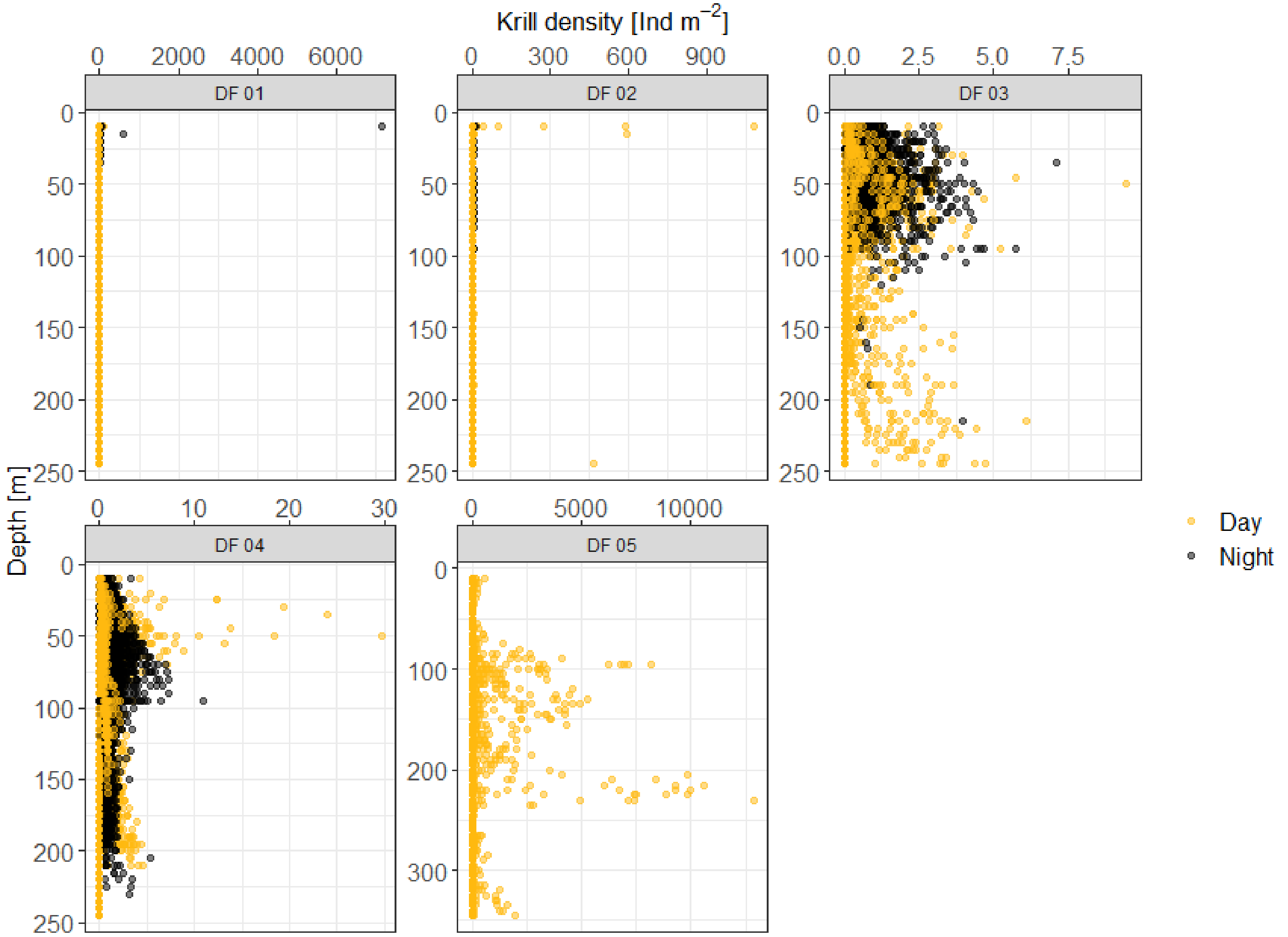

Supplementary Figure 7. Vertical distribution of Antarctic krill, *Euphausia superba*, in the top 250–300 m as obtained from the hydroacoustics survey. Krill density is given in Individuals per m² in 10 m depth bins. The panels show the density of krill for each of the drifting trap deployments (DF 1 – 5). Differences in the density of krill during day (09:00–22:00 UTC) and night (22:00–09:00 UTC) are depicted by yellow and black dots, respectively.

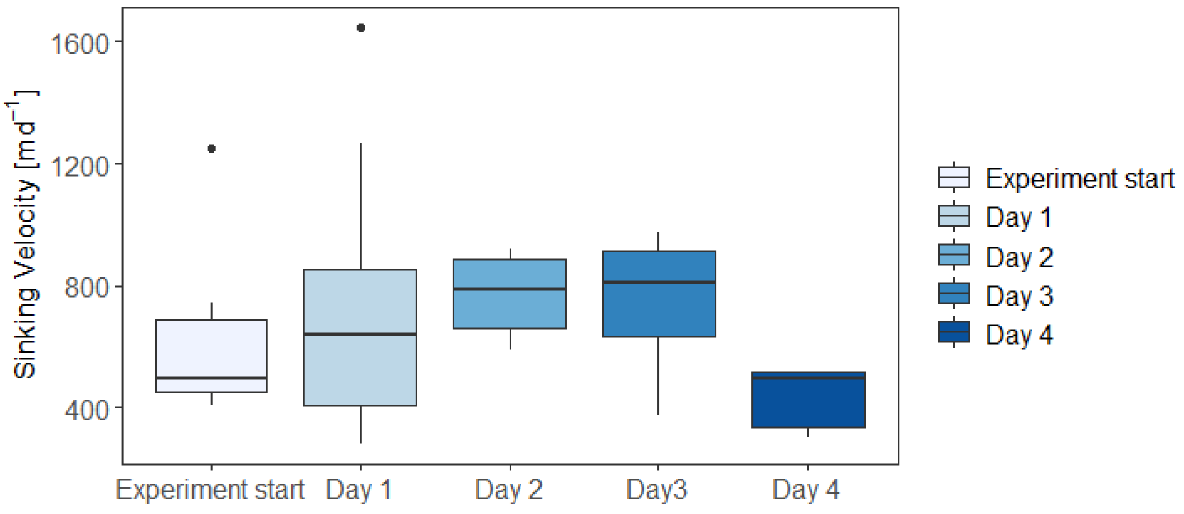

Supplementary Figure 8. Salp faecal pellet degradation experiment. Sinking velocities in m d^{-1} (y-axis) of salp faecal pellets incubated for one, two, three, and four days respectively (on the x-axis) in roller tanks mimicking the friction during the sinking process. Sample sizes (biologically independent faecal pellet samples) were n=6 for the exp. start, Day 2 and 3, respectively, n=13 for Day 1 and n=5 for Day 4. Boxplots depict the median, lower and upper hinges correspond to the 25th and 75th percentiles. The whiskers extend from the hinge to the largest value to a maximum of 1.5*IQR (interquartile range), respectively. Data points beyond 1.5*IQR are shown as single dots (outlier).

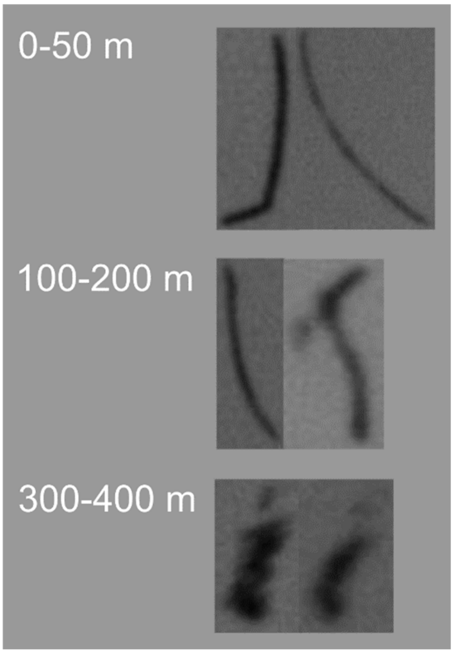

Supplementary Figure 9. Krill faecal pellet pictures obtained from the in situ particle camera at different depths, indicating fragmentation during the sinking process.

Supplementary Table 1. Deployment and recovery of all drifting sediment traps. Drifting traps were deployed at Elephant Island for five consecutive times (DF 1 – 5) between April 25 and April 30 2018. The station, date, time in UTC, latitude and longitude of each deployment and the maximum depth in meter are shown.

Drifting trap	Station	Date	Time	Latitude	Longitude	Station depth	Comment
DF 01	118_01	25-04-2018	18:53	60°59.116'S	054°57.422'W	591.4	Deployment
DF 01	118_08	26-04-2018	10:54	60°55.622'S	055°09.133'W	345.8	Recovery
DF 02	119_01	26-04-2018	15:25	60°59.765°S	054°37.844'W	571.7	Deployment
DF 02	119_15	27-04-2018	12:02	60°55.899'S	054°43.898'W	667.6	Recovery
DF 03	120_01	27-04-2018	14:06	60°56.780'S	054°45.349'W	643.7	Deployment
DF 03	120_18	28-04-2018	10:55	60°57,450'S	054°52,048'W	640.7	Recovery
DF 04	121_01	28-04-2018	13:02	60°58,114'S	054°53,030'W	632.8	Deployment
DF 04	121_13	29-04-2018	11:00	60°58.948'S	054°55.870'W	607.5	Recovery
DF 05	122_01	29-04-2018	13:03	60°59.285'S	054°57.118'W	599.5	Deployment
DF 05	122_12	30-04-2018	11:00	60°57.406'S	054°58.179'W	980.2	Recovery

Supplementary Table 2. Sampling stations for salps (*Salpa thompsoni*). All stations where salps were sampled around the study site around Elephant Island between April 21 and April 27 2018. Stations that correspond to the drifting trap deployments and which were used for the calculation of the mean salp density are marked with an asterisk.

Station	Latitude	Longitude	Day/Night	Salp abundance [Ind. m^{-2}]
116-2*	61°03.658'S	055°45.907'W	Day	266.29
119-14*	60°56.206'S	054°43.642'W	Day	12.96
120-9*	60°58.204'S	054°44.775'W	Day	2.62
107-2	61°30.070'S	053°59.324'W	Night	124.86
108-2	61°14.672'S	053°54.549'W	Night	585.77
109-2	61°00.845'S	054°00.623'W	Night	51.15
110-7	61°22.850'S	054°51.560'W	Night	95.14
114-2*	61°08.863'S	055°45.142'W	Night	183.72
119-7*	60°57.629'S	054°36.384'W	Night	296.85
119-10*	60°57.812'S	054°37.708'W	Night	356.36

Supplementary Table 3. List of all faecal pellet production experiments conducted on-board for Antarctic krill (*Euphausia superba*), and salps (*Salpa thompsoni*). The number of biological replicates (Repl.) per experiment is given. The mean size of the animals across all replicates of each experiment is given as oral-atrial length in mm for salps, and as total length in mm following the AT method by Mauchline (1980) for krill. In addition, for krill the wet weight of the animals was calculated based on Siegel's season specific length-weight relationship[6]. Faecal pellet (FP) parameters are shown as FP volume in mm^3, FP production (FPP) in mm^3 h^{-1} Ind. $^{-1}$, carbon content of FP (POC) per volume in µg mm^{-3}, FP carbon production per individual in µg h^{-1} Ind.$^{-1}$, and FP sinking velocity (SV) in m d^{-1}. Salp FP in the experiments were produced by freshly caught salps kept in surface in-situ water. Thus, the pellets referred to in this table are Type 1 FP. Type 2 salp FP were mainly observed from gel traps.

**Experiment**	**Repl.**	**Day/Night**	**Animal mean size** mm		**Weight** mg	**FP volume** mm^3		**FPP** mm^3 h^{-1} Ind. $^{-1}$		**POC** µg mm^{-3}		**POC** µg h^{-1} Ind.$^{-1}$		**SV** m d^{-1}
	Euphausia superba													
Krill Exp. 1	10	Day	29.4	± 4.8	145.87	0.09	± 0.12	0.01	± 0.01	18.52	± 24.0	2.11	± 2.6	146.53
Krill Exp. 2	8	Day	35.8	± 6.7	281.78	0.48	± 0.39	0.07	± 0.05	65.75	± 55.3	9.16	± 7.7	323.97
Krill Exp. 3	10	Day	38.3	± 3.4	353.12	1.14	± 0.32	0.13	± 0.04	126.96	± 49.6	13.99	± 5.5	-
Krill Exp. 4	10	Day	41.8	± 5.7	473.00	0.10	± 0.06	0.02	± 0.01	35.31	± 60.3	7.03	± 12.0	-
Krill Exp. 5	10	Night	41.2	± 3.9	450.69	0.34	± 0.29	0.04	± 0.03	51.33	± 39.1	4.95	± 4.32	422.37
	Salpa thompsoni													
Salp Exp. 1	8	Day	22.9	± 13.1		2.03	± 2.23	1.15	± 1.25	52.64	± 57.84	29.77	± 32.34	310.0
Salp Exp. 2	4	Night	39.3	± 15.1		2.93	± 2.23	1.03	± 0.82	76.01	± 57.79	26.68	± 21.35	765.9
Salp Exp. 3	2	Day	30.0	± 14.1		2.59	± 0.51							602.6
Salp Exp. 4	2	Night	20.0	± 0										
Salp Exp. 5	1	Day	11.0	± 0		0.60	± 0.28	0.47	± 0.22	15.52	± 7.32	12.31	± 5.81	151.6
Salp Exp. 6	1	Night	9.5	± 0		0.42	± 0.34	0.18	± 0.14	10.98	± 8.83	4.56	± 3.66	227.8
Salp Exp. 7	2	Day	15.0	± 2.8		1638.48	± 1515.60	0.44	± 0.34	46.04	± 35.52	11.45	± 8.84	777.8
Salp Exp. 8	1	Day	25	± 0		4.70	± 0			122.08	± 0			
Salp Exp. 9	1	Night	11	± 0										
Salp Exp. 10	2	Night	10.5	± 2.1		642.94	± 594.07	0.27	± 0.14	23.29	± 12.06	7.01	± 3.63	1884.3
Salp Exp. 11	3	Night	24.3	± 5.5										
Salp Exp. 12	3	Night	31.7	± 26.1										
Salp Exp. 13	2	Night	29.5	± 3.5										
Salp Exp. 14	1	Night	17	± 0										

Supplementary Table 4. List of all 38 deployments of the in-situ particle camera. The corresponding drifting traps (DF1 – 5), station, time in UTC, latitude, longitude and maximum depth of each deployment are shown.

Drifting trap	Camera profile	Station ID	Date	Time [UCT]	Latitude	Longitude	Depth [m]
DF 01	32	118_02	2018-04-25	19:47:09	- 60.9804	- 54.9623	500
DF 01	33	118_04	2018-04-26	00:06:09	- 60.9766	- 55.0232	450
DF 01	34	118_05	2018-04-26	02:03:41	- 60.9901	- 55.0206	400
DF 01	35	118_06	2018-04-26	04:02:29	- 60.9825	- 55.0214	460
DF 01	36	118_07	2018-04-26	08:02:25	- 60.9392	- 55.0711	460
DF 01	37	118_09	2018-04-26	11:41:22	- 60.9312	- 55.1617	340
DF 02	38	119_02	2018-04-26	15:37:47	- 60.9978	- 54.6088	500
DF 02	39	119_05	2018-04-26	19:10:43	- 60.9841	- 54.5528	500
DF 02	40	119_06	2018-04-26	22:04:13	- 60.96257	- 54.5623	500
DF 02	41	119_08	2018-04-27	00:22:45	- 60.96492	- 54.6229	500
DF 02	42	119_08	2018-04-27	03:03:05	- 60.97025	- 54.6111	500
DF 02	43	119_11	2018-04-27	06:04:10	- 60.95242	- 54.6303	500
DF 02	44	119_12	2018-04-27	08:00:58	- 60.93875	- 54.6544	500
DF 02	45	119_13	2018-04-27	10:01:26	- 60.93375	- 54.6837	500
DF 02	46	119_16	2018-04-27	12:42:20	- 60.93528	- 54.7316	500
DF 03	47	120_11	2018-04-27	20:06:18	- 60.95855	- 54.7553	500
DF 03	48	120_12	2018-04-28	00:05:00	- 60.93465	- 54.7690	500
DF 03	49	120_13	2018-04-28	02:01:12	- 60.94320	- 54.7807	500
DF 03	50	120_14	2018-04-28	04:04:00	- 60.96165	- 54.7669	500
DF 03	51	120_15	2018-04-28	05:58:17	- 60.96067	- 54.7666	500
DF 03	52	120_16	2018-04-28	07:57:25	- 60.96077	- 54.8178	500
DF 03	53	120_17	2018-04-28	09:51:27	- 60.95743	- 54.8675	500
DF 03	54	120_19	2018-04-28	11:35:39	- 60.96212	- 54.8764	500
DF 04	55	121_05	2018-04-28	16:46:22	- 60.96888	- 54.8769	500
DF 04	56	121_06	2018-04-28	21:59:00	- 60.98027	- 54.9071	500
DF 04	57	121_07	2018-04-29	00:00:50	- 60.98140	- 54.9153	500
DF 04	58	121_09	2018-04-29	03:00:50	- 60.98130	- 54.9228	500
DF 04	59	121_10	2018-04-29	04:58:55	- 60.98503	- 54.9031	500
DF 04	60	121_11	2018-04-29	07:00:36	- 60.98830	- 54.8837	500
DF 04	61	121_12	2018-04-29	09:02:31	- 60.98002	- 54.9087	500
DF 04	62	121_14	2018-04-29	11:39:48	- 60.98437	- 54.9375	500
DF 05	63	122_05	2018-04-29	21:03:09	- 60.96768	- 54.961	500

DF 05	64	122_06	2018-04-29	23:03:28	- 60.96767	- 54.9878	500
DF 05	65	122_07	2018-04-30	01:01:27	- 60.97360	- 55.0132	480
DF 05	66	122_08	2018-04-30	03:01:20	- 60.97287	- 55.0283	500
DF 05	67	122_09	2018-04-30	04:59:20	- 60.97413	- 55.0061	500
DF 05	68	122_10	2018-04-30	07:09:09	- 60.96278	- 54.9911	500
DF 05	69	122_11	2018-04-30	09:02:23	- 60.96418	- 54.9705	500
DF 05	70	122_13	2018-04-30	11:41:04	- 60.95763	- 54.9716	500

Supplementary Table 5. Potential faecal pellet (FP) flux of salps and the resulting export efficiency to 300 m under the regular scenario, and under the assumption that salp abundance and FP production were underestimated by 10% and 50%, respectively.

		**Regular**	**+ 10%**	**+ 50%**	**Unit**
Potential FP Flux > 170 m	Day	19.84	21.83	29.76	mg C m^{-2} d^{-1}
	Night	39.78	43.75	59.66	mg C m^{-2} d^{-1}
	24 h	59.62	65.58	89.43	mg C m^{-2} d^{-1}
Export efficiency to 300 m		19.99 ± 8.10	18.17 ± 7.36	13.33 ± 5.40	%

Supplementary References

1 Iversen, M. H. et al. Sinkers or floaters? Contribution from salp pellets to the export flux during a large bloom event in the Southern Ocean. *Deep Sea Res. Pt. II* **138,** 116-125 (2017).

2 Belcher, A. et al. The role of particle associated microbes in remineralization of fecal pellets in the upper mesopelagic of the Scotia Sea, Antarctica. *Limnol. Oceanogr.* **61,** 1049-1064 (2016).

3 Gleiber, M. R., Steinberg, D. K. & Ducklow, H. W. Time series of vertical flux of zooplankton fecal pellets on the continental shelf of the western Antarctic Peninsula. *Mar. Ecol. Prog. Ser.* **471,** 23-36 (2012).

4 Pauli, N.-C. et al. Selective feeding in Southern Ocean key grazers – diet composition of krill and salps. *Commun. Biol.* **4,** 1061 (2021).

5 Jin, X., Gruber, N., Dunne, J. P., Sarmiento, J. L. & Armstrong, R. A. Diagnosing the contribution of phytoplankton functional groups to the production and export of particulate organic carbon, $CaCO_3$, and opal from global nutrient and alkalinity distributions. *Global Biogeochem. Cycles* **20,** 1-17 (2006).

6 Siegel, V. Introducing Antarctic Krill *Euphausia Superba* Dana, 1850 in *Biology And Ecology Of Antarctic Krill* 23-41 (Springer International Publishing, Switzerland, 2016).
