# Supplementary material for: Krill and salp faecal pellets contribute equally to the carbon flux at the Antarctic Peninsula
Source: Nat Commun. 2021 Dec 9;12:7168. doi: 10.1038/s41467-021-27436-9 (PMC8660819; doi:10.1038/s41467-021-27436-9)
Supplement: Supplementary file 3 — Reporting Summary [file 41467_2021_27436_MOESM3_ESM.pdf]

nature research

Corresponding author(s): Nora-Charlotte Pauli
Bettina Meyer
Morten H. Iversen

Last updated by author(s): Oct 27, 2021

Reporting Summary

Nature Research wishes to improve the reproducibility of the work that we publish. This form provides structure for consistency and transparency in reporting. For further information on Nature Research policies, see our Editorial Policies and the Editorial Policy Checklist.

Statistics

For all statistical analyses, confirm that the following items are present in the figure legend, table legend, main text, or Methods section.

n/a	Confirmed	
[ ]	[x]	The exact sample size (*n*) for each experimental group/condition, given as a discrete number and unit of measurement
[ ]	[x]	A statement on whether measurements were taken from distinct samples or whether the same sample was measured repeatedly
[ ]	[x]	The statistical test(s) used AND whether they are one- or two-sided<br>*Only common tests should be described solely by name; describe more complex techniques in the Methods section.*
[x]	[ ]	A description of all covariates tested
[ ]	[x]	A description of any assumptions or corrections, such as tests of normality and adjustment for multiple comparisons
[ ]	[x]	A full description of the statistical parameters including central tendency (e.g. means) or other basic estimates (e.g. regression coefficient) AND variation (e.g. standard deviation) or associated estimates of uncertainty (e.g. confidence intervals)
[ ]	[x]	For null hypothesis testing, the test statistic (e.g. *F*, *t*, *r*) with confidence intervals, effect sizes, degrees of freedom and *P* value noted<br>*Give P values as exact values whenever suitable.*
[x]	[ ]	For Bayesian analysis, information on the choice of priors and Markov chain Monte Carlo settings
[x]	[ ]	For hierarchical and complex designs, identification of the appropriate level for tests and full reporting of outcomes
[x]	[ ]	Estimates of effect sizes (e.g. Cohen's *d*, Pearson's *r*), indicating how they were calculated

Our web collection on statistics for biologists contains articles on many of the points above.

Software and code

Policy information about availability of computer code

Data collection	Echo sounder SIMRAD EK60
Data analysis	Echoview, v8.0 for the analysis of hydroacoustic data, following an existing protocol by Fielding et al. 2016<br>ImageJ v1.53a for measurements of faecal pellet size<br>Matlab R2019a, Image processing toolbox, using a custom code to process images from the in-situ particle camera, following a protocol by Markussen et al. 2020<br>R, version 3.6.1 for statistical tests using the base functions<br>ggplot2, v3.3.2, R Package for visualisations

For manuscripts utilizing custom algorithms or software that are central to the research but not yet described in published literature, software must be made available to editors and reviewers. We strongly encourage code deposition in a community repository (e.g. GitHub). See the Nature Research guidelines for submitting code & software for further information.

Data

Policy information about availability of data

All manuscripts must include a data availability statement. This statement should provide the following information, where applicable:
- Accession codes, unique identifiers, or web links for publicly available datasets
- A list of figures that have associated raw data
- A description of any restrictions on data availability

All data are available within the manuscript and its supplementary material.

Field-specific reporting

Please select the one below that is the best fit for your research. If you are not sure, read the appropriate sections before making your selection.

☐ Life sciences ☐ Behavioural & social sciences ☒ Ecological, evolutionary & environmental sciences

For a reference copy of the document with all sections, see nature.com/documents/nr-reporting-summary-flat.pdf

Ecological, evolutionary & environmental sciences study design

All studies must disclose on these points even when the disclosure is negative.

Study description	The contribution of krill and salp faecal pellets to the carbon flux at Elephant Island was compared using a set of different methods. Drifting sediment traps, both traditional and equipped with a viscous gel, were deployed and accompanied by vertical profiles of an in-situ particle camera system at a high temporal resolution of about 4 hours. On-board measurements of faecal pellet production rates, faecal pellet sinking velocities and abundances of krill and salps were referenced with measurements of the carbon content of the faecal pellets and microbial respiration.
Research sample	Krill and salps are the key grazer species in the Southern Ocean and are abundant at the Antarctic Peninsula, while Elephant Island is one of the few regions were both species co-occur. We aimed at randomly sub-sampling the local populations of krill and salps that are present around the Antarctic Peninsula. The krill population at Elephant Island was dominated by female krill (63%) with only few juveniles (3.7%). The mean size of krill was 42.9 mm. The salp population was dominated by aggregate stages with a size-range from 7-20 mm and few solitary animals with a size of > 50 mm. Sampling for this study was conducted over 5 consecutive days.
Sampling strategy	Oblique net hauls to collect population parameters of krill and salps (length, sex, stage) were conducted once per station following the current regulations and recommendations by the Commission for the Conservation of Antarctic Marine Living Resources (CCAMLR). The hydroacoustic survey was conducted continuously during the entire sampling period following the relevant protocols (Fielding et al. 2016). Drifting sediment traps were deployed on five consecutive days at three depths (100, 200, 300 m) with four collection cylinder at each depth for 24 hours each. For all field sampling methods, sample size was determined by the availability of the sampling devices and deployment times of the single devices, therefore no statistical method was used.<br>Measurements of POC, chlorophyll a, faecal pellet (FP) sinking velocity, and microbial respiration were conducted in triplicates to account for within sample variation, following standard procedure for the respective instruments, therefore no additional statistical methods were conducted to assess sampling size.
Data collection	Drifting sediment traps and in-situ particle cameras were deployed on board RV Polarstern by NCP, MHI, CMF, CK with the help of the crew. Sinking particles collected with the traps were frozen and stored in sampling containers for later analyses. CN measurements were conducted on an Elemental Analyzer and data saved in Excel sheets.<br>On-board measurements of FP production, microbial respiration, and sinking velocity were collected and recorded in real time on-board and saved manually in lab protocols and excel files. Measurements of chlorophyll a, POC and primary production were collected on filters, which were frozen and stored for later analyses. Population parameters of krill and salps were recorded in real-time and saved in excel files. Hydroacoustic survey data were obtained by the Echo sounder SIMRAD EK60 and saved for later analysis using the software Echoview.
Timing and spatial scale	The spatial and temporal scale of the sampling followed the CCAMLR sampling grid along the northern Antarctic Peninsula from April 25-30 2018 at Elephant Island between 60°55.622' to 60°59.765' S and 054°37.844' to 055°09.133' W. The sampling grid aimed at replicating the AMLR grid survey (USA) to study seasonal aspects of the krill population. This grid was interrupted for five days at Elephant Island to conduct process studies on krill and salps.
Data exclusions	No data were excluded.
Reproducibility	Experimental replication was not applicable for field sampling (net hauls, hydroacoustics, drifting traps, camera profiles). Field sampling and analysis procedures were conducted following established protocols and procedures, which are stated within the manuscript and its references. Experimental replication of the faecal pellet production incubations was carried out 5 times with 10 biological replicates each. Elemental analysis of carbon and nitrogen content of the sinking particles, chlorophyll a and standing stock of POC were conducted in triplicates per sample.
Randomization	Individual krill and salps for the incubations were randomly chosen from the respective catch. Faecal pellets for measurements of sinking velocity, carbon content and sinking velocity were randomly chosen from the incubations. POC of the sediment traps was measured from a randomly chosen 1/5 split from one of three collection tubes.
Blinding	Blinding was not relevant to the field sampling as no experimental design was used. Field samples were collected without prior knowledge of sample condition. Measurements and analysis of the collected data was performed blind.

Did the study involve field work? ☒ Yes ☐ No

Field work, collection and transport

Field conditions	Ship-based sampling was conducted during calm weather conditions with mean wind forces of 5-6 Bft and sea states of about 1-2 m. Details on the field conditions during PS112 can be accessed at https://doi.org/10.2312/BzPM_0722_2018.

Location	Field work was conducted along the northern part of Elephant Island at the tip of the Antarctic Peninsula in the Southern Ocean. Exact latitudes and longitudes of each drifting trap and in-situ particle camera deployment are given in the respective tables in the supplementary material file.
Access & import/export	All field work was conducted in compliance with national and international regulations. Necessary authorization was granted by the German Environment Agency (Umweltbundesamt, UBA). The permit was granted on September 21 2017, reference number II 2.8 – 94003-3/409.
Disturbance	No particular disturbance was caused during sampling.

Reporting for specific materials, systems and methods

We require information from authors about some types of materials, experimental systems and methods used in many studies. Here, indicate whether each material, system or method listed is relevant to your study. If you are not sure if a list item applies to your research, read the appropriate section before selecting a response.

Materials & experimental systems

n/a	Involved in the study
☒	☐ Antibodies
☒	☐ Eukaryotic cell lines
☒	☐ Palaeontology and archaeology
☐	☒ Animals and other organisms
☒	☐ Human research participants
☒	☐ Clinical data
☒	☐ Dual use research of concern

Methods

n/a	Involved in the study
☒	☐ ChIP-seq
☒	☐ Flow cytometry
☒	☐ MRI-based neuroimaging

Animals and other organisms

Policy information about studies involving animals; ARRIVE guidelines recommended for reporting animal research

Laboratory animals	We did not use laboratory animals in this study.
Wild animals	Antarctic krill (Euphausia superba): 63% female adults, 33% adult male krill, 3.7% juveniles. The mean size of krill was 42.9 mm.<br>Salps (Salpa thompsoni): >50% aggregate stages (sexual generation) with a size-range from 7-20 mm, few solitary animals (asexual generation) with a size of > 50 mm.<br>Krill and salps were captured by quantitative net hauls using Isaacs-Kidd Midwater Trawls (IKMT) and Rectangular Midwater Trawls (RMT) in the upper 170 to 200 m. Immediately after the catch was on-board, animals were transferred into buckets filled with ambient water and transported to different laboratories. The majority of animals was measured, sexed, and staged and subsequently killed by being snap-frozen in liquid nitrogen for further analyses in the home laboratory. A subsample of the freshly caught animals was transported to a second laboratory, were they were transferred to experimental containers filled with ambient seawater at ambient temperature (0.5 °C) to conduct faecal pellet production incubations. Afterwards, krill and salps were killed by being snap-frozen in liquid nitrogen and stored for further analyses in the home laboratory.
Field-collected samples	Wild caught Antarctic krill and salps for faecal pellet production incubations were transferred into containers filled with seawater at ambient temperature of 0.5 °C and ambient salinity of 34.4 immediately after catch and kept in a temperature-controlled room for a maximum of 12 hours in darkness. Subsequently animals were sexed, staged and measured and snap frozen in liquid nitrogen.
Ethics oversight	No ethical approval was necessary as no vertebrates were used in this study.

Note that full information on the approval of the study protocol must also be provided in the manuscript.
